# An *In-vitro* Fracture Strength Assessment of Endodontically Treated Teeth with Different Root-end Materials

**DOI:** 10.14744/eej.2021.55265

**Published:** 2021-12-16

**Authors:** Prasanthi PENMATSA, Mohan BODDEDA, Jyothi MANDAVA, Ravichandra RAVI, Angadala PRIYANKA, Hema PULIDINDI

**Affiliations:** Department of Conservative Dentistry and Endodontics, GITAM Dental College and Hospital, Visakhapatnam, India

**Keywords:** Fracture resistance, root canal treated teeth, root-end filling materials, universal testing machine

## Abstract

**Objective:**

To assess and compare the fracture strength of endodontically treated teeth when the retrograde preparations were restored either with Biodentine or Endosequence BC RRM Fast set putty or Geristore.

**Methods:**

One hundred and twenty human mandibular premolars were used and allocated randomly into five groups (n=24 each). Following conventional root canal treatment, and apical root resection, retrograde cavities of 3 mm were prepared using ultrasonic tips. Group 1 (intact, sound teeth), Group 2 (without root-end filling), Groups 3, 4 and 5were allocated for Biodentine, Endosequence BC RRM putty, and Geristore respectively. Thermo-mechanical cyclic loading (TMC) was performed for one section of samples in each group (n=12 each) following which immediate and after TMC fracture resistance was evaluated using the Instron machine. One-way ANOVA followed by Tukey's multiple post-hoc procedures was used for data analysis.

**Results:**

Intact teeth had shown the highest fracture strength values than all other four groups (P<0.05) and resected roots without root-end filling group exhibited the lowest resistance to fracture. Amongst the test groups, Endosequence BC RRM putty displayed improved fracture strength, and Geristore exhibited the least resistance to fracture. Fracture strength values were not statistically different among Endosequence fast set putty and Biodentine group samples immediately and after thermo-mechanical cyclic loading (P=0.5987 and 0.9999 respectively). The fracture strength was notsignificantly different between Geristore and without root-end filling groups (P=0.3530).

**Conclusion:**

Endodontically treated teeth with Endosequence BC RRM putty or Biodentine root-end filled teeth had shown better fracture resistance compared to Geristore. Retrofilling with Geristore was not able to improve fracture strength of root canal-treated teeth.

## Introduction

When an endodontically treated tooth is associated with a non-healed or recurrent pathology, where orthograde retreatment procedures are not feasible, then endodontic microsurgery is considered as one valid treatment option (1). The outcome of the surgical endodontic treatment has evolved tremendously over the past decade with the use of a microscope, ultrasonic angled surgical instruments, and newer bioceramic root-end filling materials (2). Endodontic microsurgery presents a superior success rate of 94% at four years and 88% at six years compared to the 60% success rate of traditional endodontic surgical treatment (3).

**Highlights**

• Following endodontic microsurgery and retrofilling, the strength of the treated tooth is compromised with reduced length and thickness of root dentine.
• Retrofilling materials should reinforce the tooth against fracture under functional loading.
• Endosequence BCRRM putty and Biodentine retrofilled teeth were able to reinforce the root against fracture.

Though a wide range of materials are available; none of them can fulfil all ideal prerequisites of retrograde filling materials (4). Mineral trioxide aggregate (MTA) has been considered as a reference material since its inception in 1993, due to its superior antibacterial activity, marginal sealing efficacy, and ability to regenerate periodontal tissues (5). Despite the fact that MTA has a well-documented success rate as a first-generation root-end filling material, limitations such as long setting time and complex handling properties are considered as main drawbacks (6).

Biodentine (Septodont, France), a biologically active cement has mechanical properties similar to dentine. Compared to MTA, Biodentine can be handled easily with a less setting time (10 to 12 minutes),” and is dimensionally stable (7). The thickness of the interdiffusion zone formed with calcium, phosphate, and silica minerals was more for Biodentine compared to MTA, which increases over a period of time (8).

Amongst the contemporary retrograde filling materials, Endosequence bioceramic root repair material (BC RRM) available as premixed fast set putty (Brasseler, USA) was reported to have a rapid setting time (within 60 minutes) and high cell adhesion ability facilitating faster healing (9, 10). Endosequence BC RRM putty has shown increased cementum formation compared to MTA retro-filling and presented with 92% successful clinical outcome during endodontic microsurgeries (11).

Geristore (DenMat, USA) is a hydrophilic, dual-cured polyacid-modified glass ionomer cement. This material has been recommended as a root repair material for restoring resorptive lesions, perforation defects, repair of subgingival oblique fractured roots, and as a supplement to guided tissue regeneration (GTR) (12). The advantages of Geristore include low solubility and coefficient of thermal expansion, excellent bonding to the tooth, dual-cure setting mode with less curing shrinkage, high fluoride release with excellent biocompatibility, and radiopacity (13). Results of observational studies evaluating the effect of several retrograde filling materials on gingival fibroblasts manifested better cell attachment and marginal adaptation to Geristore compared to MTA (12, 14).

Following periapical surgical procedures with root-end resection and retro-preparation, root length is decreased with thinning of dentinal walls. These changes compromise the survival of the tooth due to undesirable functional stress distribution patterns leading to root fractures (15, 16). Hence, endodontically treated teeth with root-end fillings should resist the vertical fracture during the function, which is crucial for the favourable outcome of surgical endodontics.

Despite beneficial outcomes of calcium silicate materials in clinical and laboratory investigations, information on the root reinforcing capacity of these materials is limited. Moreover, it was demonstrated that cyclic functional load and thermal changes have a negative impact on the mechanical properties of root filling materials, reducing up to 30% of teeth fracture strength (17, 18). Therefore, this laboratory investigation aimed to examine the long-term fracture strength of surgical endodontically treated teeth retro-filled with different materials. The null hypothesis tested was the long-term fracture resistance of root-treated teeth is not affected by the type of retro-filling material.

## Materials and Methods

### Sample preparation

State Health University has provided approval for conducting the research under protocol no. 18D301005003 and ethical clearance was obtained from the institutional ethics committee. Calculation of the minimum sample size required was done using version 3.5, Sigmastat (Systat Software Inc, USA) software. Considering a 0.05α type error and a power of 0.80, the estimated minimum number sample for the study was one hundred. Therefore, one hundred and twenty non-carious, recently extracted mandibular premolars having single roots with single root canals were selected for the study. The length of collected teeth was standardized to 23 +/- 1 mm. While selecting these teeth, radiographs were taken in mesiodistal and buccolingual directions to ensure that all the premolar sample teeth will have similar root canal dimensions approximately. Under 2.5 X magnification, collected teeth were examined to exclude teeth with root caries, cracks, fracture lines, root resorptions, or open apices. Teeth were immersed in saline during the whole experimental period.

### Groups assignment

Random allocation of teeth into five groups was done with 24 samples each.

Group 1: Sound teeth without root canal treatment.

Group 2: Root canal treatment, apical root-resection, retro-cavity preparation without retrofilling.

Group 3: Root canal treatment, apical surgery with Biodentine root-end filling.

Group 4: Root canal treatment and retro-filling with Endosequence BC RRM fast set putty.

Group 5: Root canal treatment, apical resection followed by filling retro-cavities with Geristore.

### Root canal treatment

After preparing the access cavities for groups 2, 3, 4, and 5 teeth samples with Endo access bur (DenTsply Maillefer, Switzerland), the working length was determined radiologically. The instrumentation of the root canals was done with crown-down technique up to master apical file F3 (30/.09 variable taper) using ProTaper Gold (Dentsply, USA) rotary systems. The root canals were irrigated with 2 ml of 3% sodium hypochlorite and 3 ml of 17% EDTA for smear layer removal. Saline (5 ml) was used for final irrigation of the root canals and excess moisture was removed with absorbent points. Obturation of the root canals was done using F3 gutta-percha (Diadent, Korea) and an epoxy resin-based AH Plus (Dentsply, Germany) sealer with lateral compaction technique.

All obturated teeth were assessed radiographically to confirm the well-compacted obturation of the root canals. The access cavity restorations were done by placing a Fuji II LC liner (GC, America) and Tetric N-Ceram (Ivoclar Vivadent, USA) composite resin. Teeth were then placed in an incubator with 100% humidity at 37°C for one week, allowing the sealer to set completely.

### Root-end resection and preparation:

For all root canal treated teeth, 3mm of root-end was resected perpendicular to tooth long axis with 0°bevel using a # 702 fissure bur (SS white, NJ, USA) at high speed and copious water irrigation. Retrograde cavities were prepared to a depth of 3 mm using AS 3D surgical diamond-coated reverse angle retro-tip attached to the ultrasonic unit (Satelec, France) along with distilled water irrigation. The preparations were considered complete when there was no remaining debris or filling material on the root axial walls.

### Root-end filling:

The details of the materials used were presented in Table 1.

**Table 1.** Details of the root-end filling materials tested in the study

S. No	Root-end filling material	Composition	Lot No	Manufacturer
1	Biodentine	Powder:	B21250	Septodont, Saint
		Tri-Calcium Silicate (3Cao.Sio2),Calcium Carbonate, Calcium Oxide, Iron oxide, Zirconium Oxide and colouring agents.		Maur des Fosses, France.
		**Aqueous solution:**		
		Calcium chloride, soluble polymer(polycarboxylate).		
2	Endosequence BC RRM Fast set putty	Dicalcium silicate, tricalcium silicate, zirconium oxide, tantalum pentoxide, calcium sulfate anhydrous and filler agents.	1903FSPS	Brassler USA, Savannah, GA.
3	Geristore Syringeable	Resin:		
	Dual-cure Resin-Ionomer A2 shade	Aromatic dimethacrylate, HEMA (Hydroxyethyl methacrylate), Bis-GMA (Bisphenol A-glycidyl methacrylate), TEGDMA (Tetraethylene glycol dimethacrylate), UDMA (urethane dimethacrylate), Initiators and stabilizers. Fillers: Barium fluorosilicate glass Submicron silica.	1910000089	Den-Mat, Santa Maria, CA, USA.

BC RRM: Bioceramic root repair material

Prior to retro-filling, irrigation of the prepared cavities using sterile saline was done and dried with absorbent points. In group 3 samples, after manipulation of Biodentine, the material was placed using a messing gun (API, India), and condensed into the prepared cavities with pluggers. The root-end filled teeth were cleaned with a moistened cotton pellet and covered with wet gauze. Biodentine sets within 12 minutes from the start of mixing.

In group 4 samples, a small amount of Endosequence BC RRM fast set putty was expressed from the premixed syringe and placed into the retro-cavities using plastic filling instrument. After compacting the material with pluggers, excess material was removed with a micro brush, and it took 20 minutes for the complete set of BC RRM fast set putty.

In Geristore retro-filling group, to remove the smear layer from retro-cavities, a 2-minute application of 10% citric acid was done. After rinsing and drying the root-end cavities, the Tenure bonding agent (Brassler, USA) was applied and cured for 20 seconds. With the help of an intra-oral syringe tip, the extruded Geristore material was placed into the cavity, contoured, and light-cured for 40 seconds. The final curing time of the Geristore was 3 to 4 minutes after placing the material.

**Figure 1.**
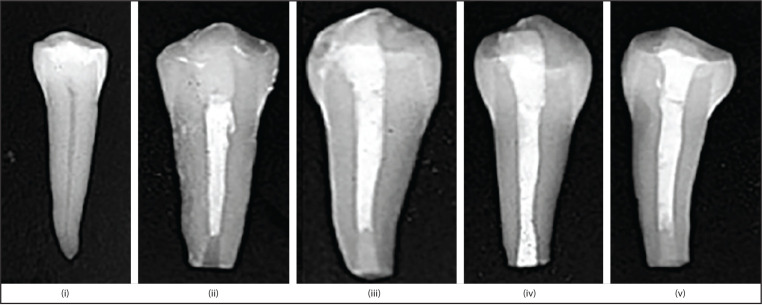
Radiographic images of (i) group 1: Sound teeth, (ii) group 2: Without root-end filling group, (iii) group 3: Biodentine, (iv) group 4: Endosequence BC RRM Fast set putty and (v) group 5: Geristore root-end filled teeth samples BC RRM: Bioceramic root repair material

After assessing the quality of root-end fillings by taking radiographs (Fig. 1), teeth were again incubated for seven days.

### Thermomechanical cyclic (TMC) loading

From all the groups, twelve samples each were thermomechanically stressed to simulate intraoral temperature changes and masticatory loads. The teeth were submitted to 10,000 thermal cycles varying between 5°C and 55°C temperature changes and one lakh mechanical cycles with an intermittent vertical occlusal load of 50N at 1 Hz frequency.

### Fracture strength evaluation

The roots of all sample teeth covered approximately with 0.3 mm of polyvinyl siloxane impression material (Prime dental products PVT Ltd, India) were embedded in acrylic auto- polymerizing resin for simulating periodontal ligament. The resistance offered by each tooth sample to fracture was assessed using a 7200 series Instron testing machine (DAK system, India). A cylindrical ball indenter having a 2.2 mm diameter was used to exert vertical compressive load at 0.5 mm/minute cross-head speed until the tooth fractured. The maximum fracture load applied in newtons was recorded for each sample, and the region of fracture on the tooth, i.e., crown or root portion or at CEJ was noted.

### Statistical analysis

The mean, standard deviation (SD), and standard error (SE) for each group was calculated. The load in newtons at which teeth fracture occurred were subjected to statistical analysis using SPSS Statistics for Windows software version 22.0 (IBM, Armonk, NY, USA). The mean fracture resistance data were analysed statistically using one-way ANOVA followed by Tukey’s posthoc procedure for pair-wise comparisons. A dependent t-test was carried out to compare immediate and after TMC fracture strength, and the location of failure was analysed using the chi-square test. Statistical analysis at 95% confidence level was performed with α=0.05 significance level.

## Results

Intact sound teeth (group 1) displayed the highest resistance to fracture, whereas group 2 samples (without retro-filling) showed the lowest fracture resistance (P=0.0001) (Table 2) among all the tested samples. Between the tested retro-filling materials, Geristore group exhibited the least fracture resistance, whereas the Endosequence BC RRM putty displayed highest resistance to fracture (P=0.0001). The Biodentine group manifested significantly better fracture resistance compared to the Geristore group (P=0.0001).

**Table 2.** Comparision of mean fracture strength (in Newtons) for all the groups

Time points	Groups	Group 1 (Sound teeth)	Group 2 (without root-end filling)	Group 3 (Biodentine)	Group 4 (Endosequence BC RRM putty)	Group 5 (Geristore)
Immediate	Mean	1274.48	646.52	909.73	958.72	723.47
	SD	121.68	70.91	103.49	59.39	58.78
	Group 1	-				
	Group 2	p=0.0001*	-			
	Group 3	p=0.0001*	p=0.0001*	-		
	Group 4	p=0.0001*	p=0.0001*	p=0.5987	-	
	Group 5	p=0.0001*	p=0.3530	p=0.0001*	p=0.0001*	-
After thermo-mechanical cycling							Mean	1152.65	534.92	734.46	739.21	607.06
SD	59.06	45.61	86.55	111.69	92.96
Group 1	-				
Group 2	p=0.0001*	-			
Group 3	p=0.0001*	p=0.0006*	-		
Group 4	p=0.0001*	p=0.0005*	p=0.9999	-	
Group 5	p=0.0001*	p=0.4953	p=0.0094*	p=0.0066*	-

*Indicates that the difference in the mean is significant at 0.05 level. BC RRM: Bioceramic root repair material, SD: Standard deviation

Among the Biodentine and Endosequence BC RRM putty groups, the difference in fracture strength was not statistically significant (P=0.5987). Statistical significance was not observed between Geristore (group 5) and without root-end filled samples (group 2) fracture strength values (P=0.3530).

**Figure 2.**
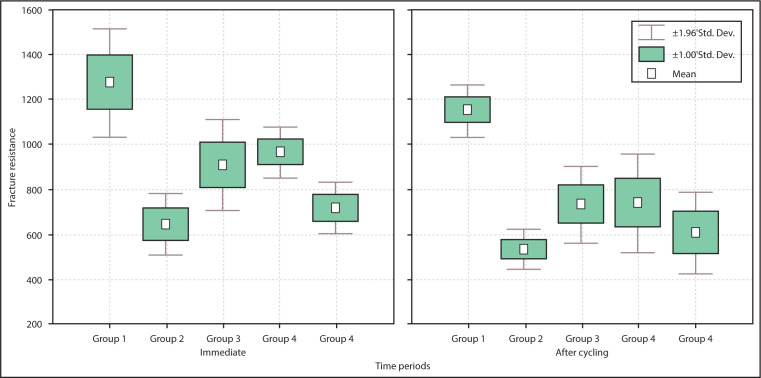
Box plots with forces required to cause fracture of teeth at different time periods

The mean fracture strength values for all the group samples disclosed significant drop after TMC (P<0.05) (Fig. 2). In response to fracture location, the difference was not significant (P=0.9900) within the groups.

## Discussion

Endodontically treated teeth have shown 50% less strength than vital teeth due to reduced dentine organic matrix and thickness leading to root fractures (19). Moreover, with apicoectomy, the altered crown to root ratio compromises the periodontal support of the tooth leading to undesirable stress distribution and susceptibility to root fracture. Hence, the root repair material selection is critical during periapical root-end surgery for successful outcomes, as these materials should be able to stimulate periradicular tissue regeneration and reinforce the root against fracture. Bioceramic materials, Biodentine and Endosequence BC RRM fast set putty, along with a modified resin ionomer cement Geristore, were selected as retrograde filling materials in this study, since all these materials have shown osteoblastic differentiation and hard tissue formation at the periradicular region (8, 11, 12).

Several studies demonstrated that cyclic functional loading and thermocycling have a negative impact on the mechanical properties of root filling materials and can cause a 20% to 30% reduction in fracture strength (17, 18). Hence, fracture strength was assessed immediately and after the aging process in the study. Mandibular premolar teeth that can simulate the clinical scenario better were used in the current study as they receive maximum masticatory forces and thus show a high prevalence for vertical root fracture (20). *In-vitro* studies covering the root surfaces with artificial periodontal ligament change the fracture mode by transferring the stresses at different root regions, and thus influence the tooth fracture strength (21). Though the applied static compressive load in this study may not simulate the intra-oral conditions completely, it is feasible to assess the reinforcing effect of different retrograde filling materials by the application of standard forces on experimental teeth (22).

According to the study results obtained, the proposed null hypothesis was rejected as the fracture resistance of the root resected teeth significantly varied according to the retrograde filling material used. In accordance with the results of several studies, (23, 24) the fracture strength was maximum for intact, sound teeth. This finding confirms that the endodontic treatment procedures lower the toughness and strength of a treated tooth.

Among the tested root-end filling materials, Endosequence BC RRM fast set putty exhibited the highest fracture strength, which was not significantly different from Biodentine group fracture resistance. These calcium silicate-based materials can form and deposit hydroxyapatite into the dentine collagen matrix by biomineralization and seals the root apex (6). Several *in-vivo* studies reported excellent periradicular healing with cellular cementum formation over the resected and retro-filled teeth with BC RRM putty (10, 11, 25). The nanoparticles (0.35μm size) of Endosequence BC RRM putty create a stronger bond to root dentine with enhanced penetration and interaction with moisture in the dentinal tubules. Having high pH with antibacterial activity, Endosequence BC RRM putty initiates alkaline phosphatase activity that increases inorganic phosphate concentration and decreases extracellular pyrophosphate inducing hard tissue formation (26). During endodontic surgical procedures, Endosequence BC RRM fast set putty can be placed and condensed easily due to its putty consistency and is less likely to be washed out before suturing (25).

Improved handling properties with faster setting time make Biodentine a suitable material for retrograde filling (8). Biodentine was reported to have the highest compressive strength (300MPa) one month after placing, similar to natural dentine (297MPa) (27). All these findings suggest that the hard tissue formation is feasible with calcium silicate materials correlating with our study results, which manifest high fracture strength values in aged samples, confirming the reinforcing effect on root resected endodontically treated teeth.

The mean resistance to fracture was significantly low for Geristore in the study, compared to calcium silicate-based root-end filling materials. Geristore is a hydrophilic dual-cure modified ionomer cement and has shown an effective seal apically in the presence of blood and moisture contaminated environment compared to MTA (14). This conflicting result in this study might be attributed to polymerization shrinkage of Geristore, causing inferior sealing with compromised fracture strength of root-filled teeth.

Regarding the location of the fracture, restorable crown fractures were observed in Biodentine and Endosequence BC RRM fast set putty samples compared to the Geristore group, though the difference was not statistically significant (P>0.05). The Geristore group exhibited more catastrophic fractures extending onto the root surface. Biodentine and Endosequence fast set putty has a modulus of elasticity closer to that of dentine with better sealing ability, which might have reduced the risk of crack propagation reinforcing the root fracture.

As new materials have been continuously introduced, it is important to gather available evidence regarding the *in-vitro* and clinical performance of these materials. More significant efforts have been made to simulate a clinical scenario in this laboratory study by performing mechanical testing procedures and acquiring standardization. However, intra-oral occlusal contact loadings during mastication cannot be accurately simulated in *in-vitro* studies. Nevertheless, Endosequence BC RRM fast set putty and Biodentine were able to reinforce the root against fracture under masticatory loads.

## Conclusion

Considering the risk of root fractures, calcium silicate-based, Endosequence BC RRM putty, and Biodentine are promising materials to be used in periapical surgical procedures with better predictable outcomes.

### Disclosures

**Conflict of interest:** The authors deny any conflict of interest.

**Ethics Committee Approval:** This study was approved by the Ethics Committee of The GITAM Dental College and Hospital Affiliated to NTR University of Health Sciences (Date: 01/04/2019, Number: 18D301005003).

**Peer-review:** Externally peer-reviewed.

**Financial Disclosure:** This study did not receive any financial support.

**Authorship contributions:** Concept – J.M., P.P., M.B., R.R.; Design – J.M., P.P., M.B.; Supervision – J.M., P.P., M.B., R.R.; Funding - M.B., R.R., A.P., H.P.; Materials - A.P., H.P., R.R.; Data collection &/or processing – J.M., P.P., M.B.; Analysis and/or interpretation – P.P., M.B., R.R.; Literature search – A.P., H.P.; Writing – M.B., P.P., J.M.; Critical Review – P.P., M.B., A.P.
